# Astrocytes in Brain Aging and Neurodegeneration: Cellular Mechanisms and Interventional Strategies

**DOI:** 10.1111/jnc.70459

**Published:** 2026-05-15

**Authors:** Flávia C. A. Gomes, Isadora Matias

**Affiliations:** ^1^ Instituto de Ciências Biomédicas Universidade Federal do Rio de Janeiro Rio de Janeiro Brazil

**Keywords:** aging, astrocyte, glial cells, neurodegenerative diseases

## Abstract

Aging is characterized by progressive changes in the physiology of brain cells, which may contribute to cognitive decline, ultimately leading to dementia and impaired quality of life. The increase in senescent cells, including glial cells in the brain, is a general feature of normal aging and has been associated with age‐related pathologies. Although recent evidence suggests that astrocytes undergo senescence in these conditions, little is known about the molecular, and cellular mechanisms underlying this event. This mini review, prepared as part of the special issue *Neurochemistry in Latin America*, provides a focused overview of astrocyte dysfunction in physiological aging and neurodegenerative conditions, integrating findings from the field alongside recent contributions from our group. We discuss how astrocyte aging contributes to cognitive decline and highlight emerging evidence on how targeting astrocytes, both genetically and pharmacologically, may rescue cognitive decline associated with aging and neurodegenerative diseases. Astrocytes produce several molecules that control synapse formation and function, which are decreased in the aging brain and in Alzheimer's disease models. In this context, recent studies indicate that astrocytes undergo significant molecular and functional remodeling during aging. Notably, astrocyte senescence has been associated with loss of lamin‐B1, nuclear alterations, impaired synaptogenic and neuritogenic capacity, altered glutamate metabolism, and mitochondrial dysfunction, all of which may contribute to reduced neuronal support and circuit integrity. In parallel, recent advances have shown that astrocyte responses during aging also include diverse reactive states that vary according to brain region, microenvironment, and disease stage. Importantly, senescence‐associated and reactive features are not mutually exclusive and may coexist or interact, further contributing to synaptic dysfunction and increased vulnerability to neurodegeneration. Finally, we discuss emerging therapeutic strategies aimed at modulating astrocyte function, including targeting astrocyte‐derived synaptogenic factors and metabolic pathways, as potential approaches to mitigate cognitive decline. Together, current evidence indicates that astrocyte dysfunction in aging reflects a complex and dynamic spectrum of cellular states that play a central role in brain vulnerability and represent promising targets for intervention in aging and neurodegenerative diseases.

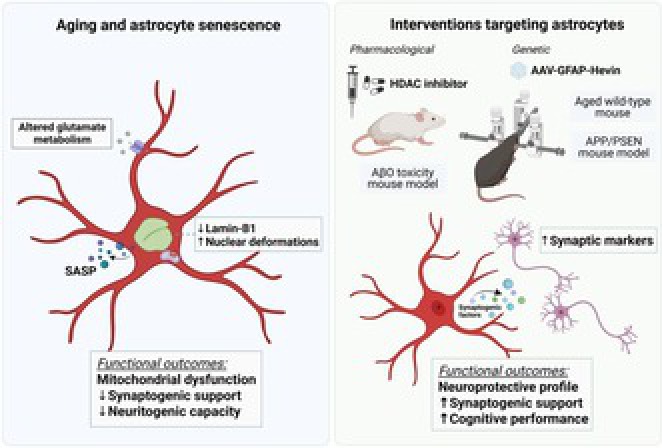

## Graphical abstract

Aging disrupts astrocyte physiology and contributes to cognitive decline. We highlight recent evidence showing that senescent astrocytes lose lamin‐B1, develop nuclear abnormalities, display impaired mitochondrial and metabolic function, and provide reduced synaptic support. These alterations are observed in vitro, in aged mice, and in the human hippocampus. We also summarize emerging strategies that target astrocytes genetically or pharmacologically, including Hevin overexpression, which enhances synaptic markers and improves cognitive performance. Together, these findings position astrocytes as promising therapeutic targets to mitigate age‐related neurodegeneration.

**Abbreviations**
AD: Alzheimer's disease
APDA: autophagy‐dysregulated astrocytes
AβOs: beta‐amyloid (Aβ) oligomers
BBB: blood–brain barrier
CNS: central nervous system
GFAP: glial fibrillary acidic protein
GS: glutamine synthetase
HDAC: histone deacetylases
iHDAC: HDAC inhibitors
iNOS: nitrogen species
NDDs: neurodegenerative diseases
PD: Parkinson's disease
ROS: reactive oxygen species
SASP: senescence‐associated secretory phenotype
SA‐βgal: β‐galactosidase

## 1 Astrocyte Heterogeneity and Vulnerability in Brain Aging

Over the past decade, Neuroscience has undergone a paradigm shift marked by a growing body of evidence positioning glial cells as central components in the functioning of the nervous system (Kettenmann et al. 2025). Originally described in 1846 by the German pathologist Rudolf Virchow as passive support cells, glial cells have gained prominence in the last two decades, especially with the discovery that they directly impact the acuity and complexity of synaptic transmission, key elements for human cognition. This scenario was further enriched by increasing evidence of their involvement in the onset and progression of several neurodegenerative diseases (NDDs) and/or neurological disorders (Diniz et al. 2017, 2019; Verkhratsky et al. 2023), highlighting glial cells as pivotal to understanding brain aging and the mechanisms that underlie age‐related vulnerability, as well as promising targets for therapeutic intervention.

Astrocytes are glial cells that control several events of brain development and function, such as providing trophic support, regulating neurotransmitter homeostasis, maintaining the blood–brain barrier (BBB), and modulating synapse formation and function (Matias et al. 2019). Beyond their supportive roles, astrocytes exhibit remarkable structural and functional diversity that varies across brain regions and under different physiological or pathological conditions. In addition to the classical distinction between protoplasmic and fibrous astrocytes in the gray and white matter, recent studies have unveiled both intra‐ and inter‐regional heterogeneity within this glial population. Currently, it is well accepted that astrocytes comprise a diverse population regarding their morphology, gene expression, and functional properties depending on their location within the central nervous system (CNS) (Bayraktar et al. 2020; Chai et al. 2017; Karpf et al. 2022), age (Boisvert et al. 2018; Clarke et al. 2018; Labarta‐Bajo and Allen 2025), and in response to different insults (Clayton and Liddelow 2025). Morphologically, they differ in soma size, process complexity, and territorial domain across cortical, hippocampal, and subcortical areas, reflecting adaptation to local neuronal architecture and synaptic organization. These regional specializations have been associated with distinct capacities for neurotransmitter clearance, metabolic coupling, and synapse formation (Batiuk et al. 2020; Bayraktar et al. 2020; Chai et al. 2017). Consistent with this, we have shown that murine astrocytes isolated from distinct brain areas display differential expression patterns of synaptogenic molecules, suggesting that their contribution to circuit assembly is inherently region‐specific (Buosi et al. 2018).

In the last decade, several of astrocytes' functions have been shown to be impaired in neuroinflammatory diseases (Diniz et al. 2017, 2019; Espírito‐Santo et al. 2021). Our group has particularly identified alterations in astrocyte pathways involved in trophic support, neurotransmitter regulation and synapse formation associated with aging and several aging‐ related diseases such as Alzheimer's and Parkinson, suggesting that astrocyte dysfunctions might be also triggers of these conditions. More recently, we have described the potential of these cells as novel therapeutic targets for Alzheimer's disease (AD) and brain aging (Cabral‐Miranda et al. 2025; Matias et al. 2022, 2023).

One of the major challenges in the clinical management of most NDDs is the lack of effective and curative therapies, not only due to the multifactorial nature of these diseases but also to the absence of reliable preclinical models that faithfully replicate the human condition, and the failure to identify effective molecular and cellular targets for drug development. Within this landscape, the 2025 pipeline for various NDDs reflects an increasing appreciation of glial cells as therapeutic targets (Cummings et al. 2025), although their full potential remains underexplored. Modulating neuroinflammation and maintaining brain homeostasis through glia, either pharmacologically or genetically, has shown promise as an alternative therapeutic strategy for NDDs (Diniz, Morgado, et al. 2024; Verkhratsky et al. 2023). In addition to emerging therapies focused on glial cells, the identification of glial biomarkers has shown potential for early diagnosis and monitoring therapeutic response in some diseases (Bellaver et al. 2025; Verkhratsky et al. 2023). However, advancing these translational strategies requires a better understanding of the cellular events that precede glial dysfunction in age‐related diseases. In this context, characterizing the early molecular and functional alterations that emerge in glial cells with age is critical to unravel the mechanisms that predispose the brain to such conditions. Indeed, transcriptomic studies have revealed that aging induces pronounced and region‐specific changes in the gene expression profiles of glial cells, particularly astrocytes, which may contribute to differential vulnerability across brain regions (Zimmer et al. 2024).

In murine models, astrocyte‐enriched transcriptomic analyses have identified region‐dependent aging signatures. Astrocytes located in the hypothalamus and cerebellum exhibit marked age‐associated shifts in gene expression, with enrichment of pathways related to synapse elimination, immune‐related processes and cholesterol homeostasis, compared to cortical astrocytes (Boisvert et al. 2018). Similarly, hippocampal and striatal astrocytes show a significant decline in transcriptional programs supporting homeostatic functions, including metabolic regulation, mitochondrial integrity and antioxidant defense, accompanied by upregulation of genes typically linked to inflammation or astrocyte reactivity (Clarke et al. 2018). In humans, multi‐regional transcriptomic analyses indicate that neuronal transcriptional profiles remain relatively conserved with advancing age, whereas glial gene expression, particularly in astrocytes and oligodendrocytes, undergoes substantial age‐related shifts, most prominently in brain regions selectively vulnerable in NDDs, including the hippocampus and substantia nigra (Soreq et al. 2017).

Astrocyte heterogeneity also encompasses distinct cellular states collectively referred to astrocyte reactivity, defined by coordinated molecular, morphological, and functional changes that arise in response to disturbances in tissue homeostasis (Escartin et al. 2021). Importantly, astrocyte reactivity is not a uniform response but instead comprises a diverse and dynamic range of states influenced by the nature of the insult, regional context, and local cellular environment (Clayton and Liddelow 2025). Initial attempts to conceptualize astrocyte reactivity proposed a classification into neurotoxic (A1) and neuroprotective (A2) states (Liddelow et al. 2017), which has since been recognized as insufficient to capture the full spectrum of astrocyte heterogeneity. Extending to aging, Clarke et al. (2018) reported the presence of A1‐like transcriptional programs in multiple brain regions. However, these findings also revealed the coexistence of alternative reactive signatures, reinforcing that astrocyte responses during aging do not conform to a strict A1/A2 dichotomy. Subsequent studies further expanded this view by identifying reactive astrocyte states linked to specific intracellular stress mechanisms. These include autophagy‐dysregulated astrocytes (APDAs), characterized by impaired autophagic–lysosomal function and reduced synaptogenic capacity during aging (E. Lee, Jung, et al. 2022), as well as disease‐associated astrocytes enriched for genes involved in endocytosis, complement activation, and lipid metabolism in neurodegenerative contexts (Habib et al. 2020). Inflammatory cues also contribute to promote reactive astrocyte states, as demonstrated by the identification of two interacting inflammatory reactive astrocyte signatures, IRAS1 and IRAS2, driven predominantly by IL‐6–STAT3 and interferon‐dependent signaling pathways, respectively (Leng et al. 2022). In parallel, astrocyte populations characterized by abnormal lipid accumulation, termed lipid‐accumulated reactive astrocytes, have been described in both human temporal lobe epilepsy and corresponding mouse models, further highlighting altered lipid handling as a feature of astrocyte dysfunction in disease (Chen et al. 2023).

Therefore, current evidence indicates that astrocyte reactivity cannot be adequately characterized by a binary A1/A2 classification, but instead reflects a spectrum of context‐dependent states (Escartin et al. 2021). Accordingly, astrocyte responses during aging and neurodegeneration encompass multiple, often overlapping profiles rather than a single reactive phenotype and may coexist with other aging‐associated astrocyte changes, including cellular senescence.

## 2 Cellular Senescence: Mechanisms and Implications for Age‐Related Diseases

Aging is a complex process characterized by physiological changes in an organism that lead to senescence and decline of biological functions. This mainly involves the progressive accumulation of cellular and tissue damage over time, leading to mitochondrial dysfunction, oxidative stress, chronic inflammation, and ultimately cognitive decline, increased dementia risk, and substantial reductions in quality of life (López‐Otín et al. 2023; Suryadevara et al. 2024).

Among the hallmarks of aging, cellular senescence is characterized by irreversible cell cycle arrest and expression of multiple molecular markers such as the tumor‐suppressor gene p16^INK4a^, elevated activity of β‐galactosidase (SA‐βgal), and the production of cytokines and proteases thus leading to a special phenotype called senescence‐associated secretory phenotype (SASP) (Cohen and Torres 2019; López‐Otín et al. 2023; Suryadevara et al. 2024). Although senescence was initially described in mitotic somatic cells, increasing evidence indicates that long‐lived, post‐mitotic cells of the CNS can also acquire senescence‐like features during aging and disease. In this review, we use the term “senescent astrocytes” to refer to astrocytes that exhibit canonical molecular and functional hallmarks of cellular senescence, despite their predominantly non‐proliferative state in the adult brain.

Within the CNS, these senescence‐associated states are mainly observed in glial populations, including astrocytes, microglia, and oligodendrocyte progenitors, although there is also evidence for post‐mitotic senescence in aged human neurons in AD brain tissue (Herdy et al. 2022). These cells progressively display molecular and morphological features of senescence, but the functional consequences of these alterations remain poorly understood. The accumulation of senescent cells during aging is associated with the onset of diseases across different tissues (Yousefzadeh et al. 2020). In the CNS, glial cell senescence represents a key component in the pathogenesis of aging‐related NDDs such as AD and Parkinson's disease (PD) (Bussian et al. 2018; Chinta et al. 2018). Therefore, the removal of senescent cells using senolytic agents, or the attenuation of SASP factors through senomorphic interventions, has emerged as a promising therapeutic avenue for age‐associated disorders. Studies in mouse models of normal aging, AD, and PD have shown that depletion of senescent cells, either genetically, by eliminating p16^INK4a^‐positive cells, or pharmacologically, through systemic administration of senolytics, significantly reduces neuroinflammation and improves cognitive and motor performance (Bussian et al. 2018; Chinta et al. 2018; Ogrodnik et al. 2021). However, the translational potential of these strategies remains limited by the lack of reliable CNS‐specific biomarkers of senescence, particularly within glial populations. Therefore, the identification of molecular signatures represents a crucial step toward refining diagnostic tools and developing targeted interventions for brain aging and neurodegenerative diseases.

Recently, our group identified a novel marker of astrocyte senescence in rodents and humans. We demonstrated that structural deformation of the nuclear lamina and reduced levels of lamin B1, a type of intermediate filament protein that composes the nuclear lamina, occur in senescent astrocytes and are associated with both murine and human aging (Matias et al. 2022).

**FIGURE 1:**
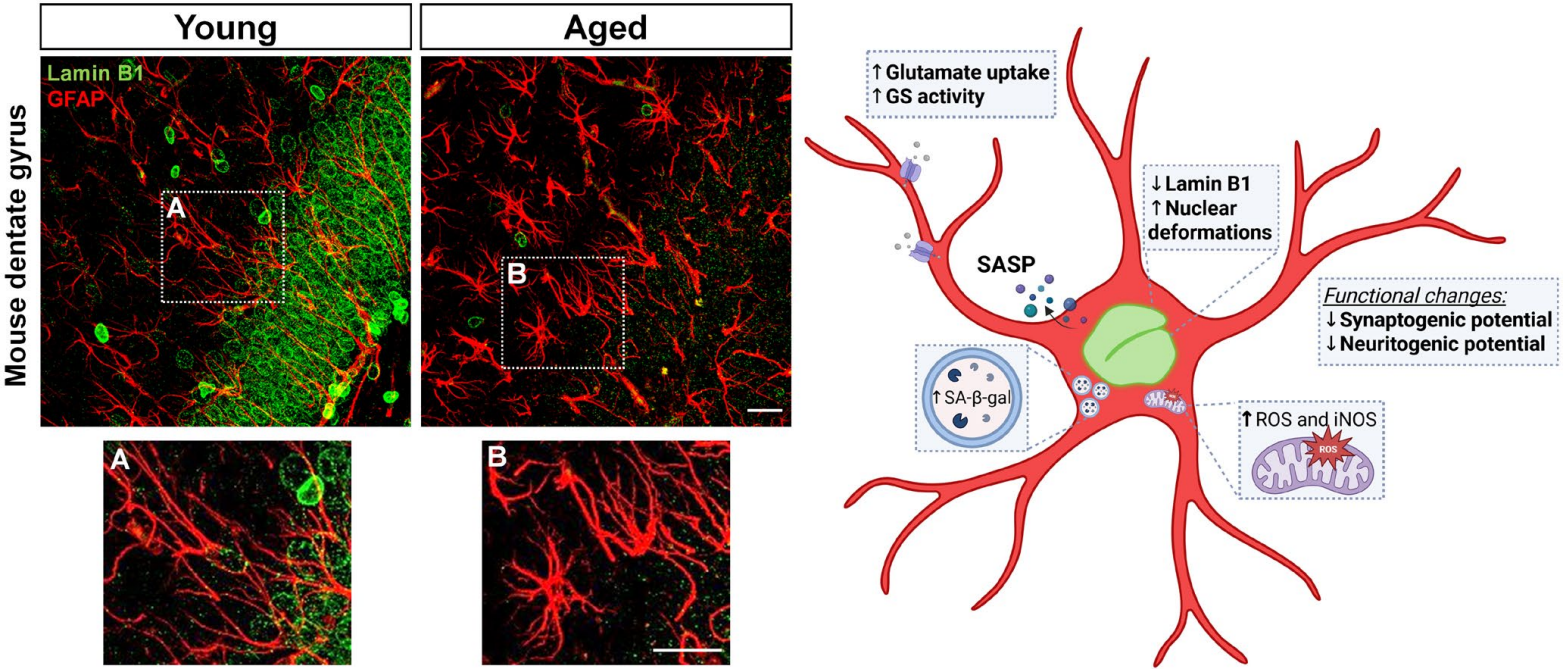
Senescent astrocyte phenotype in the aged hippocampus: From nuclear alterations to functional remodeling. Representative confocal images of the dentate gyrus from young and aged mice showing astrocytes (glial fibrillary acidic protein, GFAP, red) and lamin‐B1 (green). Aged hippocampi display a marked reduction in lamin‐B1 immunoreactivity compared to young tissue. Insets (A, B) highlight decreased lamin‐B1 intensity in aged astrocytes. The scheme on the right summarizes key features of the senescent astrocyte phenotype, including reduced lamin‐B1 expression and nuclear invaginations, increased SA‐β‐gal activity, enhanced glutamate uptake and glutamine synthetase (GS) activity, and elevated production of reactive oxygen and nitrogen species (ROS and iNOS). Functionally, senescent astrocytes exhibit reduced synaptogenic and neuritogenic potential, accompanied by the acquisition of a pro‐inflammatory SASP (senescence‐associated secretory phenotype) profile, and mitochondrial dysfunction. This schematic integrates experimental findings obtained in vitro, in murine models, and in human *post‐mortem* tissue. Schematic created using BioRender.com. Scale bars: 20 μm.

By using an in vitro model of astrocyte senescence, we observed decreased lamin B1 expression and distribution and nuclear deformations in senescent cultured astrocytes, events associated with decreased circularity of the nuclei. To characterize astrocyte senescence in vivo, we have looked for astrocyte senescence markers in the hippocampus of old mice and human brain tissue. Consistently, decrease in lamin B1 expression and nuclear deformations, as well as several other senescent markers, were also observed in the old mice hippocampus. Specifically, we found decreased lamin distribution and nuclear deformations in human neural cells from the granular cell layer of the hippocampus of *post‐mortem* brain from non‐demented elderly (Matias et al. 2022). Representative images and schematic summary of these senescence‐associated alterations based on our experimental findings are shown in Figure 1.

Evidence supporting lamin B1 loss as a feature of senescence is not restricted to astrocytes. Reduced lamin B1 expression has been consistently reported in senescent murine and human cell cultures and in multiple aging tissues, reinforcing its value as a conserved senescence‐associated marker (Freund et al. 2012; Yousefzadeh et al. 2020). Within the CNS, age‐dependent lamin B1 downregulation has also been described in adult hippocampal neural stem and progenitor cells, where it correlates with reduced proliferative capacity and impaired neurogenesis (Bedrosian et al. 2021). In line with these observations, senescent human astrocytes generated by X‐irradiation in vitro exhibit decreased lamin B1 expression together with increased levels of classical senescence markers, providing independent evidence that lamin B1 loss accompanies astrocyte senescence in human‐based models (Limbad et al. 2020).

Although the upstream mechanisms responsible for lamin B1 downregulation in astrocytes remain unclear, loss of lamin B1 has been implicated in the establishment and maintenance of senescence programs in other cell types, where it is associated with reorganization of nuclear lamina structure, changes in chromatin compartmentalization, and persistent transcriptional reprogramming (Koufi et al. 2023). In this sense, abnormalities in nuclear lamins and nuclear morphology have been reported in experimental models of age‐related NDDs, including tau‐transgenic *Drosophila* and human AD brain tissue, in which reduced lamin‐B levels and nuclear invaginations are linked to impaired cytoskeletal coupling and neuronal vulnerability (Frost et al. 2016). Similar defects in nuclear architecture have also been observed in frontotemporal dementia brain tissue and iPSC‐derived neurons, where they contribute to altered nucleocytoplasmic transport (Paonessa et al. 2019). Moreover, astrocytes from PD human brain tissue were shown to exhibit selective lamin B1 deficiency compared to neighboring cell types, suggesting that astrocytes may be particularly prone to senescence‐associated nuclear alterations in the diseased brain (Chinta et al. 2018).

Beyond nuclear structural changes, astrocyte senescence is accompanied by functional alterations that are relevant for neuronal support and circuit integrity. Our group has shown that lamin B1 reduction and nuclear deformation in astrocytes were associated with diminished synaptogenic and neurotrophic potential (Matias et al. 2022), upregulation of glutamate‐glutamine cycle activity (Matias et al. 2023), and impaired mitochondrial metabolism and biogenesis (Araujo et al. 2024; Diniz, Araujo, et al. 2024) (Figure 1).

Age‐related modulation of astrocytic glutamate‐glutamine cycle has been reported with considerable variability across studies, likely reflecting differences in experimental models, brain regions, and disease stage. While several reports describe reduced expression of glutamate transporters and impaired glutamate uptake in aged or pathological contexts (Hoshi et al. 2018; Kulijewicz‐Nawrot et al. 2013; Potier et al. 2010; Souza et al. 2015), other studies indicate preserved or increased expression of glutamate transporters and glutamine synthetase during physiological aging in rodents and humans (Clarke et al. 2018; Saransaari and Oja 1995; Wruck and Adjaye 2020). Furthermore, a recent single‐cell transcriptomic analysis has identified an aging‐associated astrocyte subpopulation that upregulates genes involved in glutamate uptake while simultaneously displaying deficits in synaptic support. APDAs described in the aged mouse hippocampus exhibit increased expression of glutamate transporter genes, including Slc1a2 and Slc1a3, together with reduced expression and secretion of synaptogenic factors, consistent with impaired astrocyte‐mediated support of synapse formation (Lee, Jung, et al. 2022). Notably, while our results demonstrate that these alterations are consistently associated with astrocyte senescence both in vitro and in aged human brain tissue, it remains unclear whether APDAs and other reactive astrocyte subpopulations described in the aged brain display classical hallmarks of cellular senescence or instead represent distinct cellular states that coexist alongside senescent astrocytes.

Collectively, these findings support the notion that glial cells are key subjects of study, not only for understanding the molecular and cellular mechanisms underlying physiological aging, but also for identifying novel aging biomarkers and molecular and cellular targets for therapeutic intervention.

## 3 Astrocyte‐Targeted Interventions in Aging and Neurodegenerative Diseases

Increasing evidence from the past decades has repositioned astrocytes from passive responders to active contributors to CNS function and dysfunction, supporting their consideration as direct therapeutic targets in aging and NDDs. As previously discussed, astrocytes undergo transcriptional, morphological and functional changes with aging, that potentially contribute to increased vulnerability to age‐related NDDs. Based on that, recent studies have emphasized the value of astrocyte phenotype and function‐oriented strategies, such as restoring astrocytic metabolic and mitochondrial competence, regulating chronic inflammation, and re‐establishing proper astrocyte‐synapse interactions, rather than broadly suppressing astrocyte activation/reactivity (Verkhratsky et al. 2023). In parallel, advances in cell‐type–targeted delivery and pharmacological modulation have strengthened the translational potential of astrocyte‐centered interventions (H.‐G. Lee, Wheeler, and Quintana 2022). Within the context of brain aging, these approaches converge on the idea that progressive dysfunction of astrocytes contributes to age‐associated vulnerability, positioning these cells as central targets for therapeutic strategies aimed at preserving tissue homeostasis and cognitive function.

In this context, we recently demonstrated that by modulating astrocyte phenotypes, either pharmacologically (Diniz, Morgado, et al. 2024) or through gene therapy (Cabral‐Miranda et al. 2025), we can reverse cognitive decline associated with aging and in a preclinical model of AD.

In the first approach, we identified a novel molecule, LASSBio‐1911, a histone deacetylase inhibitor, that reversed synaptic and behavioral impairments induced by beta‐amyloid (Aβ) oligomers (AβOs) through astrocyte modulation (Diniz, Morgado, et al. 2024).

Histone deacetylases (HDAC) are crucial enzymes involved in the regulation of gene expression through chromatin remodeling, impacting numerous cellular processes, including cell proliferation, differentiation, and survival. Recently, HDAC have emerged as therapeutic targets for NDDs, such as AD, PD, and Huntington's disease, given their role in modulating neuronal survival and plasticity and neuroinflammation. HDAC inhibitors (iHDAC) are small molecules that prevent the deacetylation of histones, thereby promoting a more relaxed chromatin structure and enhancing gene expression associated with neuroprotective pathways (Silva Franco et al. 2025). Preclinical and clinical studies have demonstrated that iHDAC can mitigate neurodegeneration, reduce neuroinflammatory markers, and improve cognitive and motor functions, positioning them as promising therapeutic agents for NDDs (Pinheiro et al. 2025).

To address the role of iHADC in AD, we have used the AβOs toxicity model. AβOs are soluble oligomers of the amyloid‐peptide that accumulate in AD brains and are considered an important toxin that lead to synaptic loss in AD. We previously found that AβOs target murine and human astrocytes, cause astrocyte activation and trigger abnormal generation of reactive oxygen species, which is accompanied by impairment of astrocyte neuroprotective potential in vitro (Diniz et al. 2017). Recently, we have shown that the iHDAC, LASSBio‐1911, mitigates the effects of AβOs on synapse loss and cognitive decline in mice. This was mainly due to its ability to modulate the astrocyte phenotype.

Whereas astrocytes from untreated animals showed molecular markers of toxicity, LASSBio‐1911 treatment reversed this phenotype, resulting in a protective profile of the glial population. Further, AβOs impair the neuroprotective potential of astrocytes (Diniz et al. 2017), while LASSBio‐1911 restores this potential (Diniz, Morgado, et al. 2024). Those data show that modulation of astrocyte phenotype and function may represent an important therapeutic alternative for NDDs, as observed by others (Clayton et al. 2024) and recently reviewed (Pinheiro et al. 2025).

Astrocytes from distinct brain regions contribute to synapse formation through the secretion of various synaptogenic molecules (Buosi et al. 2018). Among these, the extracellular matrix protein, Hevin, secreted by astrocytes, has emerged as a key player. Hevin promotes the formation of excitatory synapses by bridging pre‐ and postsynaptic proteins (Singh et al. 2016). Recent evaluation of data set from AD animal models and patients demonstrated that Hevin is downregulated in both animals and patients' brain samples (Cabral‐Miranda et al. 2025).

To explore the therapeutic potential of astrocyte‐derived Hevin in aging and AD, we employed adenovirus‐mediated overexpression of Hevin in hippocampal astrocytes of aged and APP/PS1 transgenic mice (Cabral‐Miranda et al. 2025). The use of a GFAP (glial fibrillary acidic protein) gene promoter ensured astrocyte‐specific expression of the transgene. Behavioral analyses revealed that Hevin overexpression not only ameliorated cognitive, synaptic, and behavioral deficits in APP/PS1 mice, but also improved performance in aged wild‐type animals.

To elucidate the underlying molecular mechanisms, we performed proteomic profiling of hippocampal tissue from Hevin‐overexpressing animals. The analysis revealed significant modulation of proteins involved in synaptic transmission, cognition, and dendritic development–further supporting the role of astrocyte‐derived Hevin in maintaining synaptic integrity (Cabral‐Miranda et al. 2025). In summary, our findings demonstrate that overexpression of Hevin in astrocytes modulates synaptic pathways and improves cognitive function in both aged and AD model mice. These results highlight the therapeutic potential of targeting astrocyte‐secreted synaptogenic factors for mitigating cognitive decline associated with aging and NDDs.

Recently, therapeutic strategies targeting cellular senescence have emerged as a complementary avenue for astrocyte‐targeted interventions in brain aging and NDDs. Genetic or pharmacological elimination of p16^INK4a^‐positive senescent glial cells have been shown to attenuate neuroinflammation, preserve neuronal integrity, and improve cognitive and motor outcomes in mouse models of aging, AD, and PD, providing causal evidence that senescent glia actively contribute to CNS dysfunction (Bussian et al. 2018; Chinta et al. 2018). Beyond cell elimination, senomorphic approaches aimed at attenuating the senescent phenotype have gained particular relevance in the CNS, where preservation of post‐mitotic cells is relevant. In this context, modulation of astrocyte senescence through metabolic and mitochondrial pathways has proven effective in restoring astrocytic homeostatic functions and conferring neuroprotection, as demonstrated by rapamycin‐induced mTOR inhibition and by metformin‐mediated suppression of astrocytic cGAS–STING signaling (Araujo et al. 2024; Wang et al. 2024). In parallel, emerging immunotherapeutic strategies, such as CAR‐T cells engineered to recognize senescence‐associated surface markers, have provided proof‐of‐concept evidence that senescent astrocytes can be selectively targeted with high specificity, opening new perspectives for precision senotherapy in the aging brain (Deng et al. 2024).

Taken together, the studies discussed in this section support the view that manipulating astrocytic phenotype, whether through functional reprogramming or selective clearance/attenuation of senescent cells, represents a promising strategy for both neurodegenerative and age‐associated cognitive decline while also highlighting the need for careful consideration of cell‐type specificity, timing, and safety in future translational efforts.

## 4 Conclusions and Future Directions

Taken together, our data from the last decade, along with accumulating evidence from the literature, suggest that astrocyte alterations and dysfunctions during aging may constitute triggers in the onset and progression of various neuropathological conditions. These changes likely contribute to increased brain vulnerability to aging and other insults. As discussed, both reactive and senescent astrocytes exhibit profound transcriptional and metabolic reprogramming, leading to disruptions in homeostatic functions, secretory profiles, and neuron–glia communication. However, it remains unclear whether sustained astrocyte reactivity can evolve into a senescence‐like state or, conversely, whether senescent astrocytes can promote glial reactivity, forming a pathological positive feedback loop. Future studies integrating single‐cell and spatial transcriptomics, together with functional manipulation, will be instrumental in defining the causal hierarchy between astrocyte reactivity, senescence, and neurodegeneration, as well as how the regional specificity of these phenotypes contribute to brain vulnerability/resilience. Understanding how these states intersect, whether as sequential or overlapping processes, will be crucial to identify intervention points capable of halting or reversing glial‐driven neurodegeneration. Therefore, elucidating the molecular mechanisms underlying astrocyte dysfunctions and exploring their potential as therapeutic targets may pave the way for novel diagnostic and interventional strategies for age‐associated neurological disorders.

## Author Contributions

**Flávia C. A. Gomes:** conceptualization, writing – original draft, writing – review and editing, project administration, funding acquisition. **Isadora Matias:** conceptualization, writing – review and editing, visualization, funding acquisition.

## Funding

Works from the group are supported by grants from Conselho Nacional de Desenvolvimento Científico e Tecnológico (CNPq) (Flávia C. A. Gomes), Ministério da Saúde, Departamento de Ciência e Tecnologia (MS‐DECIT) (Flávia C. A. Gomes), Fundação Carlos Chagas Filho de Amparo à Pesquisa do Estado do Rio de Janeiro (FAPERJ) (Flávia C. A. Gomes, Isadora Matias) and Instituto Nacional de Ciência & Tecnologia da Glia (INCT‐Glia) (CNPq) (Flávia C. A. Gomes).

## Conflicts of Interest

Flávia C.A. Gomes is a member of the Financial and Advisory Committee of the International Society for Neurochemistry (ISN), and Chair of the ISN Flagship School Committee.

## Data Availability

The authors have nothing to report.
